# Identification of a morphogene required for tapered filament termini in filamentous cyanobacteria

**DOI:** 10.1099/mic.0.001416

**Published:** 2023-11-16

**Authors:** Gabriel A. Parrett, Peyton D. Brones, Garrett M. Jenkins, Savanna M. Mounts, Alicia Nguyen, Douglas D. Risser

**Affiliations:** ^1^​ Department of Biology, University of Colorado Colorado Springs, Colorado Springs, CO 80918, USA

**Keywords:** cyanobacteria, motility, morphogene, cell morphology, phenotypic plasticity

## Abstract

Although the photosynthetic cyanobacteria are monophyletic, they exhibit substantial morphological diversity across species, and even within an individual species due to phenotypic plasticity in response to life cycles and environmental signals. This is particularly prominent among the multicellular filamentous cyanobacteria. One example of this is the appearance of tapering at the filament termini. However, the morphogenes controlling this phenotype and the adaptive function of this morphology are not well defined. Here, using the model filamentous cyanobacterium *Nostoc punctiforme* ATCC29133 (PCC73102), we identify *tftA*, a morphogene required for the development of tapered filament termini. The *tftA* gene is specifically expressed in developing hormogonia, motile trichomes where the tapered filament morphology is observed, and encodes a protein containing putative amidase_3 and glucosaminidase domains, implying a function in peptidoglycan hydrolysis. Deletion of *tftA* abolished filament tapering inidcating that TftA plays a role in remodelling the cell wall to produce tapered filaments. Genomic conservation of *tftA* specifically in filamentous cyanobacteria indicates this is likely to be a conserved mechanism among these organisms. Finally, motility assays indicate that filaments with tapered termini migrate more efficiently through dense substratum, providing a plausible biological role for this morphology.

## Introduction

Oxygenic photosynthesis first evolved in cyanobacteria, the only prokaryotic organisms capable of this metabolic process. Although cyanobacteria are monophyletic, they display substantial diversity in their morphology [1]. Individual species of cyanobacteria possess spherical or rod-shaped cells and can be unicellular or multicellular, the latter growing in long chains of cells that are linear or branching [1]. As has been shown for other bacteria, the rod-shaped morphology observed in cyanobacteria is dependent on orthologs of the elongasome complex, such as the actin homolog MreB and associated proteins, which typically coordinate the sites of cell wall synthesis to produce rod-shaped cells (for review see [2]). Curiously, elongasome orthologs are nearly ubiquitous in cyanobacteria, even in many strains that display coccoid rather than rod-like cell morphologies [2]. Among the filamentous strains, tapering of one or several cells at the end of filaments is also frequently observed, so that one or both ends of the filament come to a point [1]. The biological function and genes involved in tapered filaments have yet to be elucidated.

While many species display static morphologies over their life cycle, others, particularly those among the filamentous cyanobacteria, are also capable of substantial phenotypic plasticity, meaning that the cell and filament morphologies can change dramatically over time depending on their life cycle and environmental stimuli [2]. One example of this is the transition between spherical and rod-shaped cells observed during complementary chromatic acclimation (CCA) in *Fremyella diplosiphon* [3]. Illumination with red light leads to the activation of kinase activity by RcaE, which in turn promotes transcription of *bolA* [4–6]. BolA is a transcriptional repressor which then binds to the promoter of *mreB* repressing its transcription, leading to spherical cells [6]. Under illumination with green light RcaE activity is suppressed, causing *bolA* expression to be reduced, resulting in enhanced *mreB* expression and a transition to rod-shaped cells [6].

A second example of this phenotypic plasticity is the development of specialized motile filaments termed hormogonia. Hormogonium development is commonly observed in heterocyst-forming cyanobacteria, and like CCA, involves a transition from more spherical vegetative cells to more rod-shaped cells in hormogonia [7]. In *N. punctiforme *, where some details about the hormogonium gene regulatory network (GRN) have been revealed, the transition to rod-shaped cells also appears to be driven primarily by modulating expression of elongasome genes. Hormogonium development is initiated by the hybrid histidine kinase HrmX (formerly HrmK) [7, 8] which in turn activates transcription of a hierarchical sigma factor cascade [9] where induction of *sigJ* expression subsequently leads to upregulation of *sigC* and *sigF*. SigJ also directly promotes expression of elongasome genes such as the *mreBCD* locus and *rodA* [9]. In *N. punctiforme* and many other hormogonium-forming cyanobacteria, tapering of filaments at the termini is observed exclusively in hormogonia [1], indicating that the morphogenes involved are likely to be regulated by the hormogonium GRN. Given the appearance of the tapered filament termini exclusively in motile filaments, one logical hypothesis is that this morphology plays a role in enhancing motility by functioning as a wedge, allowing the filaments to better bypass obstacles and traverse dense substratum.

In this study, a combination of genetic, comparative genomic and transcriptomic approaches was employed to identify a novel morphogene required for tapering in filamentous cyanobacteria and show that this morphology enhances motility in dense substratum.

## Methods

### Strains and culture conditions

For a detailed description of the strains used in this study refer to Table S1. *N. punctiforme* ATCC 29133 and its derivatives were cultured in Allan and Arnon medium diluted four-fold (AA/4), without supplementation of fixed nitrogen, as previously described [10], with the exception that 4 and 10 mM sucralose was added to liquid and solid medium, respectively, to inhibit hormogonium formation [11]. For hormogonium induction for phenotypic analysis, the equivalent of 30 μg ml^−1^ chlorophyll *a* (Chl *a*) of cell material from cultures at a Chl *a* concentration of 10–20 μg ml^−1^ was harvested at 2 000 ***g*** for 3 min, washed two times with AA/4 and resuspended in 2 ml of fresh AA/4 without sucralose. For selective growth, the medium was supplemented with 50 μg ml^−1^ neomycin. *Escherichia coli* cultures were grown in lysogeny broth (LB) for liquid cultures or LB supplemented with 1.5 % (w/v) agar for plates. Selective growth medium was supplemented with 50 μg ml^−1^ kanamycin, 50 μg ml^−1^ ampicillin, and 15 μg ml^−1^ chloramphenicol.

### Plasmid and strain construction

For a detailed description of the plasmids, strains, and oligonucleotides used in this study refer to Table S1, available in the online version of this article. All constructs were sequenced to insure fidelity.

To construct plasmid pDDR467 for in-frame deletion of *tftA*, approximately 900 bp of flanking DNA on either side of the gene and several codons at the beginning and end of the gene were amplified via overlap extension PCR using primers NpR3584-5′-F, NpF3584-5′-R, NpF3584-3′-F and NpF3584-3′-R, and cloned into pRL278 [12] as a BamHI-SacI fragment using restriction sites introduced on the primers.

To construct plasmid pDDR542, a mobilizable shuttle vector containing *tftA* and its putative promoter region, the coding-region and 5′ region 200 bp upstream of the TSS [13] (301 bp upstream of the start codon) were amplified via PCR using primers PNpF3584-F and NpF3584-R and subsequently cloned into pAM504 [14] as a BamHI‐SacI fragment using restriction sites introduced on the primers.

To construct plasmid pDDR561 for expression recombinant TftA-6xHis in *E. coli *, the coding-region of *tftA* was amplified via PCR using the primers NpF3584-NcoI-F and NpF3584-XhoI-R and subsequently cloned into pET28a as an NcoI-XhoI fragment using restriction sites introduced on the primers.

Gene deletion was performed as previously described [15] with *N. punctiforme* cultures supplemented with 4 mM sucralose to inhibit hormogonium development and enhance conjugation efficiency [11, 16]. To construct UOP166, plasmid pDDR467 was introduced into wild-type *N. punctiforme* ATCC29133.

### Purification of recombinant TftA-H6 and zymogram assays

Plasmid pDDR561 was transformed into *E. coli* BL21(DE3). Transformants were grown at 37 °C in liquid cultures to an OD_600_ of ~0.6 and subsequently, expression of recombinant TftA-6xHis was induced by the addition of 1 mM isopropyl β-D-1-thiogalactopyranoside (IPTG) for 5 h. Cells were harvested and stored at −20 °C. Purification of TftA-6xHis was performed using the Ni-NTA Spin Kit (Qiagen) following the manufacturers protocols for purification under native conditions, with the exception that lysis of the cells was performed using a probe sonicator (ten rounds of 10 s followed by 10 s on ice to prevent overheating the sample), as enzymatic lysis using lysozyme might interfere with subsequent zymogram assays. Eluted fractions were analysed on a 12 % SDS-PAGE gel followed by Coomassie staining. Zymogram assays were performed as previously described [17].

### Motility assays

To determine the ability of strains to migrate through varying concentrations of agar, plates containing AA/4 medium and noble agar at concentrations of either 0.5 %, 1 %, or 1.5 % were first prepared. A sterile inoculating needle was then used to plunge a colony of *N. punctiforme* below the surface of the agar, and 50 µl of molten agar of the same concentration as the respective plate was pipetted over the lesion in the surface of the agar and allowed to solidify so that hormogonia would be forced to migrate through the solidified medium rather than migrating through the open lesion to the surface of the plate. Plates were subsequently incubated for 1 week and then the colony was divided arbitrarily into four quadrants and the furthest distance a filament had migrated from the inoculation site in each quadrant was measured. This was repeated for all four quadrants and averaged to determine the maximal migration distance for each colony, and the entire experiment was repeated in triplicate.

### Microscopy

Light microscopy was performed with an EVOS M5000 fluorescence microscope (Life Technologies) equipped with a 40× objective lens. To quantify the tapering of terminal cells, the width of 50 terminal cells for each strain was measured at the base of the cell, adjacent to the next cell in the filament, and then at the mid-cell. To calculate the tapering index, the width at the mid-cell was then subtracted from the width at the base. Measurements were conducted on three biological replicates. For fluorescent vancomycin (Van-FL, Invitrogen cat# V34850) labelling of cell walls, vegetative filaments were induced to form hormogonia and 6 h following induction, 1 µl of Van-FL dissolved in dimethyl sulfoxide (1 µg µl^−1^) was added to 1 ml of culture. The culture was subsequently incubated for an additional 6 h, the time period during which morphological changes to the cell morphology occur during hormogonium development. Following labelling, the cells were washed two times with AA/4, and Van-FL was subsequently imaged using an EVOS M5000 fluorescence microscope (Life Technologies). Excitation and emission were as follows: EVOS light cube, GFP (AMEP4651: excitation 470±22 nm, emission 525±50 nm) for Van-FL and EVOS Light Cube, RFP (AMEP4652: excitation 531±40 nm, emission 593±40 nm) for cellular autofluorescence.

## Results

### Identification of a putative morphogene specific to filamentous cyanobacteria

**Fig. 1.**
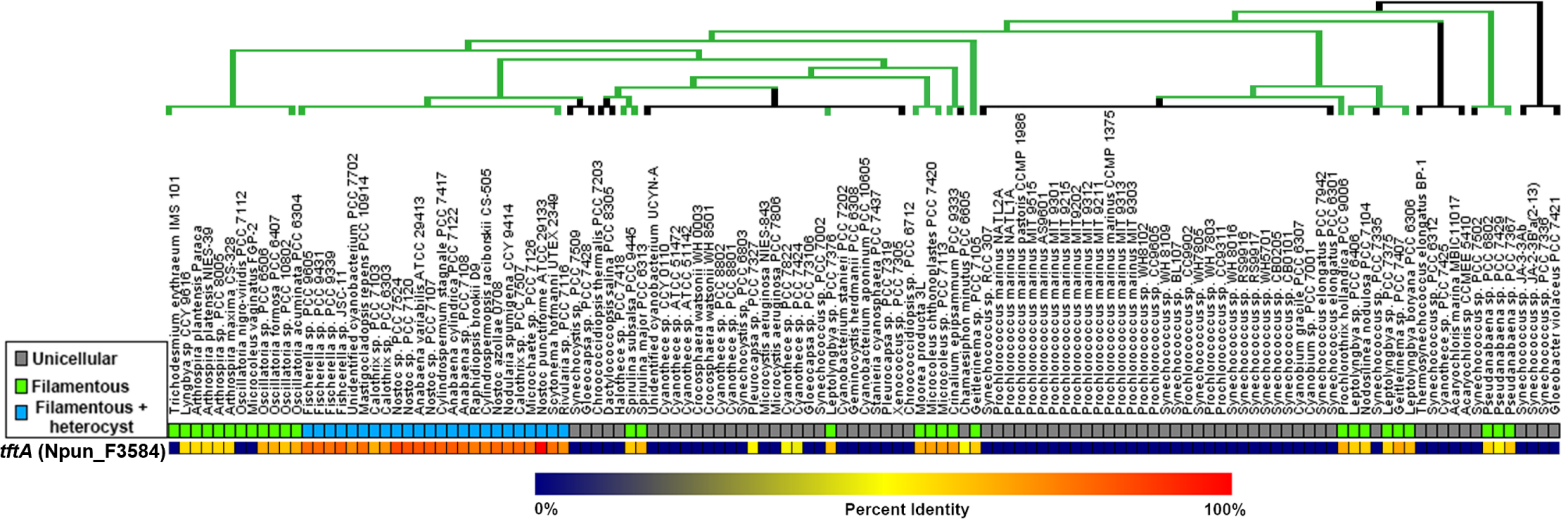
Evolutionary conservation of *tftA*. Heat map depicting the percent identity for orthologs of *N. punctiforme tftA* in cyanobacteria, derived from data reported by Cho *et al*. [18]. Species organization and phylogenetic tree based on the phylogeny reported by Shih *et al*. [30], but depicting the finding, as reported by Schirrmeister *et al*. [31], that most extant cyanobacteria are derived from a filamentous ancestor. For the phylogenetic tree, green=filamentous, black=unicellular.

Given that the tapered filament morphology is widespread in filamentous cyanobacteria [1], limited to hormogonia in *N. punctiforme* [7], and likely to involve remodelling of the cell wall, we searched the *N. punctiforme* genome for genes that are enriched specifically in filamentous cyanobacteria, upregulated in hormogonium development, and encode protein domains associated with cell wall synthesis or degradation. This search led to the identification of a single candidate gene, locus tag Npun_F3584, as a potential morphogene responsible for tapered filaments, which we have designated *tftA* (tapered filament termini A) based on the results presented below. Using a previously generated comparative genomics data set of cyanobacterial genomes subjected to a hierarchical clustering analysis [18, 19], we manually searched for clusters of genes primarily found exclusively in filamentous cyanobacteria, as opposed to unicellular species. This led to the initial identification of *tftA*. Orthologs of *tftA* were encoded in the genomes of ~94 % of filamentous strains investigated, while only present in ~4 % of unicellular cyanobacteria (Fig. 1).

**Fig. 2.**
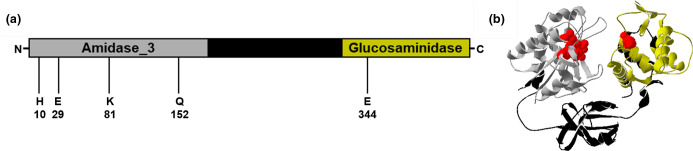
Predicted structure of TftA. (**a**) Schematic diagram depicting the domains of TftA and the putative active sites for the amidase_3 and glucosaminidase domains. (**b**) A ribbon diagram depicting the putative tertiary structure of TftA based on Alphafold, coloured according to the predicted domains as indicated in (**a**). Putative active site residues depicted as space filling model in red. Image generated using Swiss-PdbViewer 4.1.0 [32].

TftA is annotated as a cell wall hydrolase/autolysin and is predicted to encode both amidase_3 (pfam01520) and glucosaminidase (pfam01832) domains (Fig. 2a). Amidase_3 domains cleave the bond between N-acetylmuramic acid and the peptides which cross-link peptidoglycan strands [20], while glucosaminidase domains hydrolyse the glycosidic bonds in polysaccharides containing N-acetylglucosamine [21, 22]. The amidase_3 domain is found at the N-terminus of TftA while the glucosaminidase domain is at the C-terminus, separated by 138 amino acids of unknown function (Fig. 2a). The putative structure of TftA based on AlphaFold [23] indicates that this region of unknown function serves as a linker primarily composed of a β-sheet, which connects the two discrete functional domains (Fig. 2b). The putative active site residues for the amidase_3 domain [24] based on sequence alignments (Fig. S1) are in close proximity in the predicted structure, although conservative substitutions at K81 and Q152 have replaced the histidine and glutamate residues typically found in these positions (Fig. 2a, b). The active site residues for glucosaminidase domains are not characterized as well, but at minimum are thought to involve a glutamate [25], and in sequence alignments TftA shares an absolutely conserved glutamate residue with other known glucosaminidases (Fig. S2) that is likely to be part of the active site (Fig. 2a, b). This data indicates that TftA may facilitate degradation of peptidoglycan and thus be involved in cell wall remodelling.

To experimentally validate that TftA exhibits hydrolytic activity towards peptidoglycan, a zymogram assay [17] was attempted. Recombinant, 6 x histidine tagged TftA was expressed in *E. coli* and subsequently enriched by nickel affinity chromatography. Due to the extremely low expression of TftA in *E. coli* the TftA enrichment was highly impure (Fig. S3). However, a clear band at the expected molecular weight for TftA was present in samples where expression was induced by the addition of IPTG, and absent in a control sample where IPTG was excluded (Fig. S3). These enrichments were subsequently used to perform zymogram assays where cell wall material is included in the PAGE gel, and hydrolytic activity results in a zone of clearing for proteins exhibiting hydrolytic activity. An extremely faint zone of clearing was observed at the expected molecular weight for TftA, but this was very difficult to image (Fig. S3). Thus, the results from this experiment were ultimately inconclusive and future studies will be needed to demonstrate that TftA is a bona fide peptidoglycan hydrolase.

**Fig. 3.**
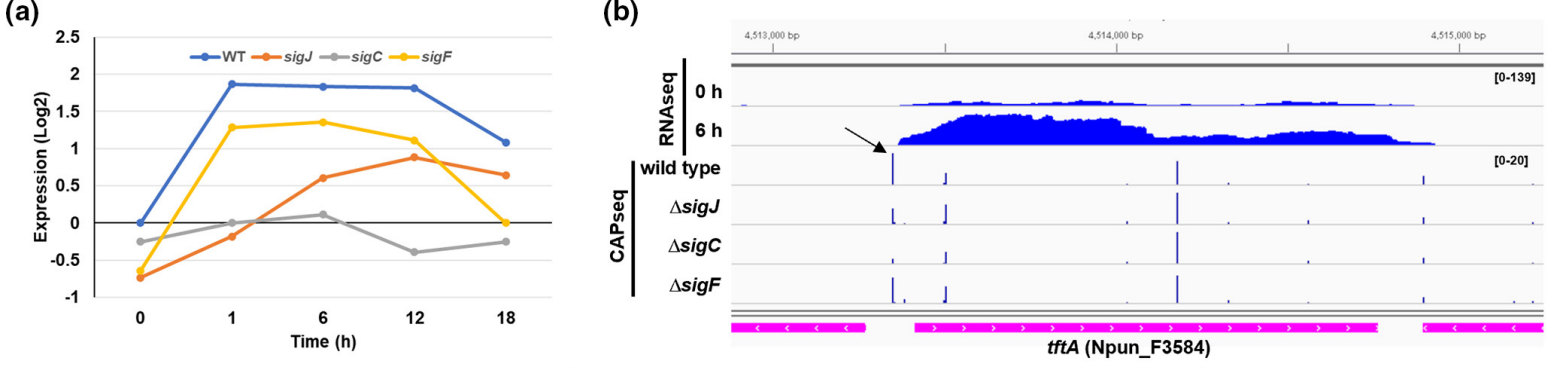
Expression of *tftA* in developing hormogonia. (**a**) Graph depicting the expression of *tftA* in developing hormogonia of the wild-type and hormogonium-specific sigma factor mutants 0–18 h post-hormogonium induction. Expression=Log2(experimental strain and time point/wild-type t=0). (**b**) Read map coverage from RNAseq and CAPseq data for various strains and time points as indicated. Read map coverage values for RNAseq and CAPseq indicated in brackets. Arrow indicates position of SigC-dependent TSS.

Hormogonium development is driven, in part, by a hierarchical sigma factor cascade [9]. This cascade is initiated by upregulation of *sigJ*, which in turn promotes transcription of *sigC* and *sigF*. Each of the three sigma factors subsequently promotes transcription of a regulon of hormogonium-specific genes. Expression of *tftA* in developing hormogonia, based on a previously generated RNAseq dataset [9], indicated enhanced transcription, which was almost completely abolished in a Δ*sigC* strain and diminished to a lesser extent in a Δ*sigJ* mutant (Fig. 3a). Given that SigJ directly enhances transcription of *sigC* [9], these results indicate that upregulation of *tftA* in developing hormogonia is most directly dependent on SigC. Read coverage from RNAseq and genomic context indicate that *tftA* is monocistronic (Fig. 3b) and transcriptional start site (TSS) mapping using an available Cappable-seq data set (CAPseq) [13] indicated the presence of a transcriptional start site in the *tftA* promoter region that was substantially reduced in the Δ*sigC* strain (Fig. 3b), further supporting the hypothesis that *tftA* expression is dependent on SigC. Collectively, the genomic conservation, predicted protein function, and transcriptional regulation indicate that *tftA* fulfils the necessary criteria to be a candidate gene involved in the production of tapered filaments.

### tftA is essential for the formation of tapered filament termini

**Fig. 4.**
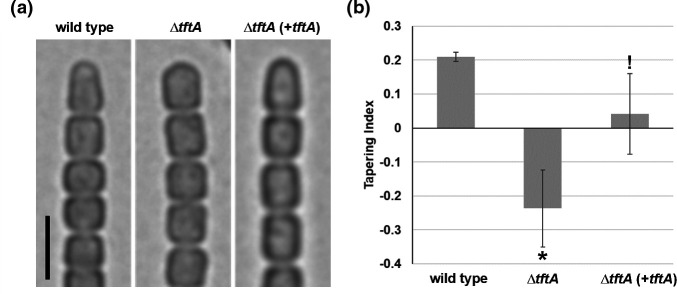
Phenotype of the Δ*tftA* strain. (**a**) Light micrographs of the filament termini for the wild-type, Δ*tftA*, and Δ*tftA* strain harbouring a plasmid expressing *tftA* from its native promoter (+*tftA*) as indicated. Black bar=5 µm. (**b**) Quantification of filament tapering. Strains as described in (**a**). Tapering index was calculated by subtracting the width of the mid-cell from the width of the base of the cell adjacent to the adjoining cell in the filament. Error bars = ± 1 S.D. * = *p*-value<0.01 as determined by two-tailed Student’s t-test between the wild-type and Δ*tftA* and Δ*tftA* (+*tftA*) strains; != *p*-value<0.05 as determined by two-tailed Student’s t-test between the Δ*tftA* and Δ*tftA* (+*tftA*) strains, *n*=3.

To experimentally validate the involvement of *tftA* in the formation of tapered filaments, an in-frame deletion strain of *tftA* was generated (Fig. S4). Compared to the wild-type strain, hormogonia of the Δ*tftA* strain produced much blunter cells at the filament termini (Fig. 4a, b), but otherwise had no obvious effect on hormogonium morphology. To confirm that deletion of *tftA* was responsible for the observed phenotype, a replicative shuttle vector encoding *tftA* expressed from its native promoter was introduced into the Δ*tftA* strain. The introduction of this plasmid partially restored the tapered filament morphology confirming that the phenotype was due to deletion of *tftA* (Fig. 4a, b). We also noticed that cultures of the complementation strain grew poorly and contained many lysed cells, possibly due to enhanced expression of *tftA* from the multi-copy replicative plasmid and the resultant increase in peptidoglycan hydrolysis activity of TftA. These results support the hypothesis that TftA plays a role in producing the tapered filament termini phenotype.

To further investigate the hypothesis that TftA is involved in remodelling of the cell wall at the filament termini, we attempted to employ fluorescent vancomycin (Van-FL) labelling to visualize differences in peptidoglycan synthesis at the filament termini in the wild-type and Δ*tftA* strain during hormogonium development (Fig. S5). However, while both strains tended to accumulate more Van-FL at the filament termini, especially at the apical tip, where a bright fluorescent focus was often present, no obvious difference in Van-FL incorporation was observed between the strains. The presence of the bright foci observed in both strains is likely the result of rapid peptidoglycan synthesis to repair the lesion at the site where fragmentation of the filament occurred during hormogonium development. Given that TftA is predicted to be involved primarily in the removal of old peptidoglycan, rather than the incorporation of new peptidoglycan, pulse-chase experiments utilizing Van-FL might prove a better approach to observe differences in cell wall remodelling. However, Van-FL labelling in vegetative cells was very weak and rapidly lost throughout filaments following hormogonium induction, thus making this approach unfeasible.

### Tapered filaments enhance migration in dense substratum

**Fig. 5.**
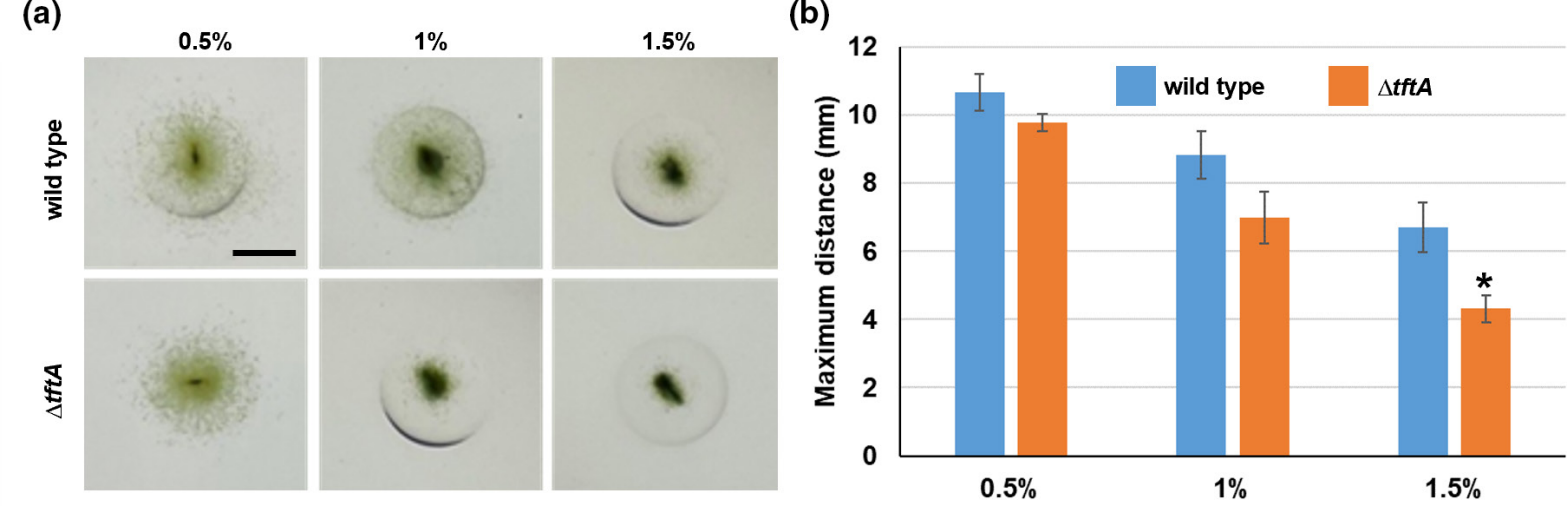
Motility assays in the wild-type and Δ*tftA* strain. (**a**) Images of colonies for the wild-type and Δ*tftA* strain (as indicated) migrating through varying concentrations of agar (as indicated). Black bar=5 mm. (**b**) Quantification of colony spreading through varying concentrations of agar. Error bars = ±- 1 S.D. * = *p*-value<0.05 as determined by two-tailed Student’s t-test between the wild-type and Δ*tftA* at each corresponding agar concentration, *n*=3.

One hypothesis to account for the presence of tapered filament termini is that this aids in the migration of filaments through their environment, particularly in dense substratum where the tapered ends can function as a wedge. To test this hypothesis, motility assays were conducted where wild-type and Δ*tftA* hormogonia were forced to migrate through various concentrations of agar (Fig. 5a, b). In media containing 0.5 % agar, the distance of migration for each strain was similar, with a slight decrease for the Δ*tftA* strain that was not statistically significant. However, as the agar concentration increased to 1 and 1.5% the discrepancy in migration between the strains became more pronounced, with the Δ*tftA* strain only exhibiting ~2/3 the migration distance of the wild-type strain in 1.5 % agar. Therefore, while *tftA* and tapered filament termini are dispensable for motility, these results support the hypothesis that tapered filament termini enhance the migration of hormogonia through dense substratum.

## Discussion

As with other bacteria, the shape of cyanobacterial cells is largely determined by the architecture of the cell wall. For example, the appearance of rod-shaped cells in cyanobacteria is dependent on the elongasome, a complex comprised of the cytoskeletal protein MreB, among others, which coordinates the site of peptidoglycan synthesis to establish rod-shaped morphology, and deletion of the gene for the cell wall hydrolase AmiC2 in *N. punctiforme* has been shown to dramatically alter cell and filament morphology and disrupt cell differentiation [17]. Furthermore, studies of other bacteria with non-canonical cell shapes have implicated the activity of cell wall hydrolases in their unique morphologies (for review, see [26]). The results presented here support the hypothesis that *tftA* is a morphogene responsible for producing the tapered filament morphology. Given the predicted function, it is likely that TftA facilitates this transition by selectively hydrolysing the peptidoglycan at the apical end of terminal cells to facilitate cell wall remodelling, although further experimental evidence is needed to confirm that TftA exhibits hydrolytic activity. The near absolute conservation of *tftA* in filamentous cyanobacteria indicates that this represents a conserved mechanism in most filamentous species. It is currently unclear how TftA selectively facilitates the remodelling of the cell wall specifically at the filament terminus. Attempts to determine the spatial expression of *tftA* along the filament and subcellular localization of TftA within cells via the construction of a *tftA-gfp* translational fusion strain did not produce visible fluorescence. We suspect that this is likely due to the extremely low level of *tftA* expression based on read coverage from RNAseq (Fig. 3b). Thus, more work is needed to determine how the activity of TftA is spatially limited within the cells and filaments. It is also unclear if *tftA* alone is sufficient to produce tapered termini, or if additional genes are involved in this process.

The results from this study also indicate that the presence of tapered filaments provides a selective advantage when migrating through dense substratum. Previous studies have demonstrated that the genes required for surface motility in *N. punctiforme* are widely conserved among filamentous cyanobacteria [9, 18, 19, 27, 28], and that a subset of these genes are confined to filamentous species [28], similar to the conservation pattern of *tftA* reported here. Therefore, we propose that the tapered filament phenotype was an adaptation that evolved to enhance the ability of filamentous cyanobacteria to move through dense substratum, such as the microbial mats through which filamentous cyanobacteria are known to migrate [29]. Like medium containing higher concentrations of agar, microbial mats are composed of extra polymeric substances densely populated with cells and would therefore lead to high rates of collisions for filaments migrating through them. Tapered filaments would presumably reduce the rate at which such collisions would stall motility.

## Supplementary Data

Supplementary material 1
