# Supplementary material for: Identification of a morphogene required for tapered filament termini in filamentous cyanobacteria
Source: Microbiology (Reading). 2023 Nov 16;169(11):001416. doi: 10.1099/mic.0.001416 (PMC10710843; doi:10.1099/mic.0.001416)
Supplement: Supplementary material 1 [file mic-169-1416-s001.pdf]

```
TftA            321 KLYREEAGIEGVNYDIAFCQMCVETGFL      R.[2].GDIKPE      QNNFAGL.[ 4].GGSEA       ASFPSA 379
WP_003502324     12 GYAISDMKENGILASVTIAQAILESSWG       T      SELAKK      ANNYFGM.[13].DRVSK.[21].RAYPDI 98  [Clostridium] symbi...
CCY16106         40 GLAVEQMERYGIPASITLAQGLLESAAG       Q      SELSRK      ANNHFGI.[ 6].NGPYV.[11].RAYSSP 109 Prevotella sp. CAG:755
WP_014224736     39 HLAVEQEKKYHIPASIKLAQAILESDAG       R      AKLARE      ANNHFGI.[ 6].KGKKT.[11].RTYGSV 108 Tannerella forsythia
Q03GX3          711 ADIVNVCNSNKLYASVAMAQAVLESGYG       T      SSLAQE      AHNLFGI.[ 6].KGATY.[19].RKYASI 788 Pediococcus pentosa...
EQC78962        171 ESARKIGQKYDLYASVMIAQAILESASG       Q      SQLAQA.[1].NYNLFGI.[ 4].NGNFV.[20].RVYENY 248 Enterococcus sp. HS...
Q1GBZ0           48 LQAQKTSKKYGVYASLMLAQAALESGWG       T      STLSTQ      ANNFFGM.[ 5].TGATY.[20].RKYSSY 125 Lactobacillus delbr...
WP_012402305    172 APAQAASAATGIPARFIIGQAALESGWG.[4].R      KSDGST      SHNIFGI.[ 6].TGKTV.[19].RAYDSY 253 Paraburkholderia ph...
jgi:Lreu_1812    53 PAAQREQKKYHIPASITIAQAGLESNWG       R      SRLANK      YNNLFGI.[ 4].DDEKV.[19].TVYNSW 128 Lactobacillus reute...
WP_003502324     12 GYAISDMKENGILASVTIAQAILESSWG       T      SELAKK      ANNYFGM.[13].DRVSK.[21].RAYPDI 98  [Clostridium] symbi...
CCY16106         40 GLAVEQMERYGIPASITLAQGLLESAAG       Q      SELSRK      ANNHFGI.[ 6].NGPYV.[11].RAYSSP 109 Prevotella sp. CAG:755
WP_014224736     39 HLAVEQEKKYHIPASIKLAQAILESDAG       R      AKLARE      ANNHFGI.[ 6].KGKKT.[11].RTYGSV 108 Tannerella forsythia
Q03GX3          711 ADIVNVCNSNKLYASVAMAQAVLESGYG       T      SSLAQE      AHNLFGI.[ 6].KGATY.[19].RKYASI 788 Pediococcus pentosa...
EQC78962        171 ESARKIGQKYDLYASVMIAQAILESASG       Q      SQLAQA.[1].NYNLFGI.[ 4].NGNFV.[20].RVYENY 248 Enterococcus sp. HS...
Q1GBZ0           48 LQAQKTSKKYGVYASLMLAQAALESGWG       T      STLSTQ      ANNFFGM.[ 5].TGATY.[20].RKYSSY 125 Lactobacillus delbr...
WP_012402305    172 APAQAASAATGIPARFIIGQAALESGWG.[4].R      KSDGST      SHNIFGI.[ 6].TGKTV.[19].RAYDSY 253 Paraburkholderia ph...
jgi:Lreu_1812    53 PAAQREQKKYHIPASITIAQAGLESNWG       R      SRLANK      YNNLFGI.[ 4].DDEKV.[19].TVYNSW 128 Lactobacillus reute...
```

```
TftA            380 RIGVRAH 386
WP_003502324     99 EMSIKDH 105 [Clostridium] symbiosum
CCY16106        110 RESYEDH 116 Prevotella sp. CAG:755
WP_014224736    109 TDSYEDH 115 Tannerella forsythia
Q03GX3          789 KDSIADY 795 Pediococcus pentosaceus ATCC 25745
EQC78962        249 EESFEDY 255 Enterococcus sp. HSIEG1
Q1GBZ0          126 QASFDDY 132 Lactobacillus delbrueckii subsp. bulgaricus ATCC 11842 = JCM 1002
WP_012402305    254 EEAMTDY 260 Paraburkholderia phymatum
jgi:Lreu_1812   129 ADSINAH 135 Lactobacillus reuteri DSM 20016
WP_003502324     99 EMSIKDH 105 [Clostridium] symbiosum
CCY16106        110 RESYEDH 116 Prevotella sp. CAG:755
WP_014224736    109 TDSYEDH 115 Tannerella forsythia
Q03GX3          789 KDSIADY 795 Pediococcus pentosaceus ATCC 25745
EQC78962        249 EESFEDY 255 Enterococcus sp. HSIEG1
Q1GBZ0          126 QASFDDY 132 Lactobacillus delbrueckii subsp. bulgaricus ATCC 11842 = JCM 1002
WP_012402305    254 EEAMTDY 260 Paraburkholderia phymatum
jgi:Lreu_1812   129 ADSINAH 135 Lactobacillus reuteri DSM 20016
```

Figure S1. Sequence alignment between the amidase_3 domain of TftA and other amidase_3 domain-containing proteins. Putative active site residues are highlighted yellow and indicated with #.

```
Feature 1                       #                        #
  TftA           3 RIFISAAHGG.[6].DSGAIA.[ 2].TTEAKEMILLRDLIVTELR.[3].LE      ILAV.[ 5].AAQTITWI 67
1XOV_A           4 NYSMSRGHSD.[1].CVGAED.[ 1].LSEIKEAEKVLNAASDELK.[3].HN.[1].KTFI.[10].LNKIVNWH 68  Listeria phage PSA
YP_001253940     2 KIGIDCGHTL.[3].DYGAVG.[ 1].KAESNLTREVGTKVISKLQ.[3].HT.[1].VKCY.[10].LSYRANTA 68  Clostridium botulinum A ...
YP_429849      120 VVVIDPGHGG.[1].DPGHRG.[ 3].LLERDMAWKTAGELARILE.[3].AR.[1].VMTR.[ 7].WQERLARV 183 Moorella thermoacetica A...
YP_510478      171 VVMLDPGHGG.[1].DPGALR.[ 2].FSEAELILTFARELREVMR.[4].IE.[1].QMTR.[ 7].LPTRVTLA 234 Jannaschia sp. CCS1
NP_972318      114 VILIDPGHGG.[1].DPGCVG.[10].LYEKDIALKVSLDLYKMLK.[5].KK.[1].LLTR.[ 7].LEDRVNMA 186 Treponema denticola ATCC...
YP_001876261   303 KIVIDPGHGG.[1].DPGATR.[ 3].STEKDINLWIAKELYALLK.[3].FD.[1].KLTR.[ 7].LNQRSKIS 366 Elusimicrobium minutum P...
ZP_02990456    314 TVVIDAGHGG.[1].DPGAVY.[ 2].VYEKVIALDIAKRLAGILS.[4].YN.[1].KLTR.[ 7].LEQRVSLN 377 Exiguobacterium sp. AT1b
ZP_00055854     21 LIALDVGHYQ.[1].APGEIS.[ 3].VPELVFNTELARRVGWELD.[3].IA.[1].TLIN.[ 7].LAERPRRA 84  Magnetospirillum magneto...
1XOV_A           4 NYSMSRGHSD.[1].CVGAED.[ 1].LSEIKEAEKVLNAASDELK.[3].HN.[1].KTFI.[10].LNKIVNWH 68  Listeria phage PSA
YP_001253940     2 KIGIDCGHTL.[3].DYGAVG.[ 1].KAESNLTREVGTKVISKLQ.[3].HT.[1].VKCY.[10].LSYRANTA 68  Clostridium botulinum A ...
YP_429849      120 VVVIDPGHGG.[1].DPGHRG.[ 3].LLERDMAWKTAGELARILE.[3].AR.[1].VMTR.[ 7].WQERLARV 183 Moorella thermoacetica A...
YP_510478      171 VVMLDPGHGG.[1].DPGALR.[ 2].FSEAELILTFARELREVMR.[4].IE.[1].QMTR.[ 7].LPTRVTLA 234 Jannaschia sp. CCS1
NP_972318      114 VILIDPGHGG.[1].DPGCVG.[10].LYEKDIALKVSLDLYKMLK.[5].KK.[1].LLTR.[ 7].LEDRVNMA 186 Treponema denticola ATCC...
YP_001876261   303 KIVIDPGHGG.[1].DPGATR.[ 3].STEKDINLWIAKELYALLK.[3].FD.[1].KLTR.[ 7].LNQRSKIS 366 Elusimicrobium minutum P...
ZP_02990456    314 TVVIDAGHGG.[1].DPGAVY.[ 2].VYEKVIALDIAKRLAGILS.[4].YN.[1].KLTR.[ 7].LEQRVSLN 377 Exiguobacterium sp. AT1b
ZP_00055854     21 LIALDVGHYQ.[1].APGEIS.[ 3].VPELVFNTELARRVGWELD.[3].IA.[1].TLIN.[ 7].LAERPRRA 84  Magnetospirillum magneto...

Feature 1                     #
  TftA          68 .[ 7].DVALEIKSDA.[ 6].RGASVFY.[3].N.[ 6].GEQL     LVGLLRRVP.[ 1].LPNR     GVKP 129
1XOV_A          69 .[ 5].DVHISVHLNA.[ 3].TGVEVWY     Y.[ 5].GRKL.[3].ISAKMAKAL.[ 1].LPNR     GAKA 124 Listeria phage PSA
YP_001253940    69 .[ 5].DLYVSIHFNC.[ 5].HGTEVLT     Y.[ 3].SFTE.[3].VLNNICALG      YTNR     GIKD 123 Clostridium botulinum ...
YP_429849      184 .[ 4].FCFLSIHEYG.[ 6].RGTAVLY     N.[ 5].NEEL.[3].VLERIVTRV.[ 1].TPPR     GTSP 241 Moorella thermoacetica...
YP_510478      235 .[ 5].DLFISLHADA.[ 6].QGATVYT     L.[49].SRAF.[3].LVQAIDMAG.[ 2].LHAR     PRGE 338 Jannaschia sp. CCS1
NP_972318      187 .[10].ILYVSIHVNA.[ 6].AGFEVWY     L.[33].SILM.[3].ILDGLDAQI.[ 3].SPNR     GIRE 280 Treponema denticola AT...
YP_001876261   367 .[ 5].DLFVSIHANA.[ 6].QGFEVYF     R.[47].SSKL.[3].VRNSVKATA.[ 6].NPNS     SIKQ 472 Elusimicrobium minutum...
ZP_02990456    378 .[ 5].DVFVSLHANS.[ 6].HGHEVLV     P.[13].SRSL.[3].INKEIASRI.[ 2].IQNR     GVKY 445 Exiguobacterium sp. AT1b
ZP_00055854     85 .[ 5].SLFLSLHHDS.[22].SGFGLFV     S.[ 8].SLRV.[3].IADSLLVQG.[12].GENR.[7].GIYR 180 Magnetospirillum magne...
1XOV_A          69 .[ 5].DVHISVHLNA.[ 3].TGVEVWY     Y.[ 5].GRKL.[3].ISAKMAKAL.[ 1].LPNR     GAKA 124 Listeria phage PSA
YP_001253940    69 .[ 5].DLYVSIHFNC.[ 5].HGTEVLT     Y.[ 3].SFTE.[3].VLNNICALG      YTNR     GIKD 123 Clostridium botulinum ...
YP_429849      184 .[ 4].FCFLSIHEYG.[ 6].RGTAVLY     N.[ 5].NEEL.[3].VLERIVTRV.[ 1].TPPR     GTSP 241 Moorella thermoacetica...
YP_510478      235 .[ 5].DLFISLHADA.[ 6].QGATVYT     L.[49].SRAF.[3].LVQAIDMAG.[ 2].LHAR     PRGE 338 Jannaschia sp. CCS1
NP_972318      187 .[10].ILYVSIHVNA.[ 6].AGFEVWY     L.[33].SILM.[3].ILDGLDAQI.[ 3].SPNR     GIRE 280 Treponema denticola AT...
YP_001876261   367 .[ 5].DLFVSIHANA.[ 6].QGFEVYF     R.[47].SSKL.[3].VRNSVKATA.[ 6].NPNS     SIKQ 472 Elusimicrobium minutum...
ZP_02990456    378 .[ 5].DVFVSLHANS.[ 6].HGHEVLV     P.[13].SRSL.[3].INKEIASRI.[ 2].IQNR     GVKY 445 Exiguobacterium sp. AT1b

Feature 1                                      #
  TftA         130 .[6].G.[1].LAFCRQ.[3].PALAMQVGFLSNPEDRALLQ.[2].RRDFALGIADGLA 181
1XOV_A         125      T.[2].LRFLNS.[3].TAVLLEVCFVDRKEDANAIH.[4].YDKLGIAIAEGLT 173 Listeria phage PSA
YP_001253940   124      G.[2].LYVLKH.[3].KAMIIECCFCDNRNDMNRYN     AENMANAIVKGLV 168 Clostridium botulinum A str. ATCC 3502
YP_429849      242      D.[2].LVQLGQ.[1].PALRIEPVTITNWVDEGLLR.[4].HQKTALAVVVAIK 288 Moorella thermoacetica ATCC 39073
YP_510478      339      G.[1].FSVLKA.[3].PAVLLEIGFLSEGGDLENIQ.[4].RAQMQAAITDAVL 386 Jannaschia sp. CCS1
NP_972318      281      N.[1].WFVVRN.[3].PSVLIELGFISNKTEIKLLN.[4].LKKCSLGIYNGLS 328 Treponema denticola ATCC 35405
YP_001876261   473      A.[1].FYVLKG.[3].PAILVECGYISNPSDRKQLN.[4].RNKLAEGIYKGIL 520 Elusimicrobium minutum Pei191
ZP_02990456    446      Q.[2].YVVGRN.[2].PSTLVEYGFISNSSDRSYLT.[4].RQRMAEATASGIH 493 Exiguobacterium sp. AT1b
ZP_00055854    181      F.[2].LAVLRQ.[3].PAVLIEAGIIVNRDDEVLIA.[4].RAAFARAITAAAA 229 Magnetospirillum magnetotacticum MS-1
1XOV_A         125      T.[2].LRFLNS.[3].TAVLLEVCFVDRKEDANAIH.[4].YDKLGIAIAEGLT 173 Listeria phage PSA
YP_001253940   124      G.[2].LYVLKH.[3].KAMIIECCFCDNRNDMNRYN     AENMANAIVKGLV 168 Clostridium botulinum A str. ATCC 3502
YP_429849      242      D.[2].LVQLGQ.[1].PALRIEPVTITNWVDEGLLR.[4].HQKTALAVVVAIK 288 Moorella thermoacetica ATCC 39073
YP_510478      339      G.[1].FSVLKA.[3].PAVLLEIGFLSEGGDLENIQ.[4].RAQMQAAITDAVL 386 Jannaschia sp. CCS1
NP_972318      281      N.[1].WFVVRN.[3].PSVLIELGFISNKTEIKLLN.[4].LKKCSLGIYNGLS 328 Treponema denticola ATCC 35405
YP_001876261   473      A.[1].FYVLKG.[3].PAILVECGYISNPSDRKQLN.[4].RNKLAEGIYKGIL 520 Elusimicrobium minutum Pei191
ZP_02990456    446      Q.[2].YVVGRN.[2].PSTLVEYGFISNSSDRSYLT.[4].RQRMAEATASGIH 493 Exiguobacterium sp. AT1b
ZP_00055854    181      F.[2].LAVLRQ.[3].PAVLIEAGIIVNRDDEVLIA.[4].RAAFARAITAAAA 229 Magnetospirillum magnetotacticum MS-1
```

Figure S2. Sequence alignment between the glucosaminidase domain of TftA and other glucosaminidase domain-containing proteins. Putative active site residues are highlighted yellow.

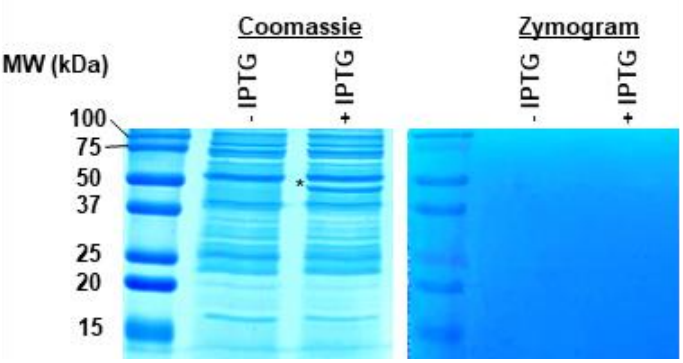

Figure S3. Zymogram assays of recombinant TftA-6xHis. TftA-6xHis was enriched by nickel-affinity chromatography from *E. coli* either induced for expression of TftA-6xHis with 1 mM IPTG (+IPTG), or with IPTG omitted (-IPTG) as a negative control. Eluted fractions were subsequently analyzed by SDS-PAGE on a 12% Gel and Coomassie stained for protein visualization or subjected to zymogram assays. *indicates a band at the expected molecular weight for TftA (48.89 kDa) enriched in IPTG induced samples but absent in the negative control where IPTG is omitted.

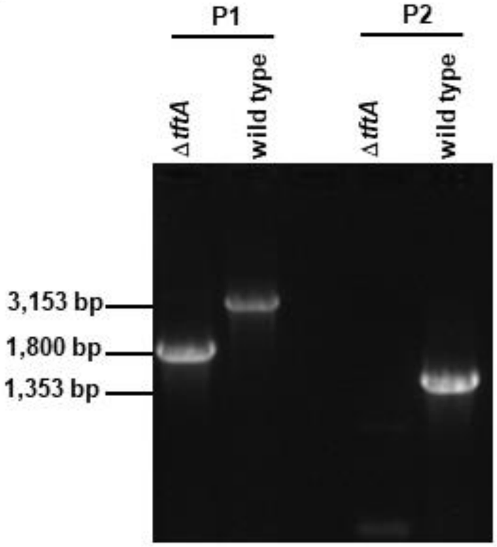

Figure S4. Confirmation of the mutant genotype for the Δ*tftA* strain. PCR reactions were performed using chromosomal DNA from the wild type, or Δ*tftA* strain (as indicated), using either primers NpF3584-5'-con-F and NpF3584-3'-con-R (P1) that anneal distal to the regions of homology incorporated on plasmid pDDR467 for generation of the deletion strain, or primers NpF3584-NcoI-F and NpF3584-XhoI-R (P2) that anneal at the 5' and 3' end of the *tftA* gene. The P1 reaction produced fragments of the expected molecular weight for both the wild type (3,153 bp) and Δ*tftA* strain (1,800 bp). The P2 reaction produced a fragment at the expected molecular weight for *tftA* (1,353 bp) for the wild type but failed to yield a product for the Δ*tftA* strain, confirming it is isogenic and *tftA* has been fully deleted from the genome.

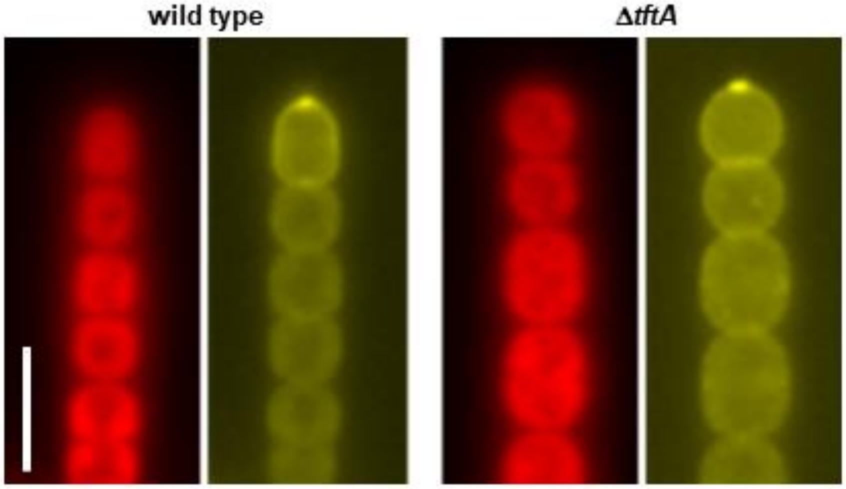

Figure S5. Van-FL labeling of filament termini in developing hormogonia. Fluorescence micrographs of the wild type and Δ*tftA* strain labeled with Van-FL 12 h post hormogonium induction. Autofluorescence is depicted in red and Van-FL fluorescence in yellow. White bar=5μm.

Table S1. Strains, Plasmids, and Primers

**strain name**	**description**
N. punctiforme ATCC29133	wild type
UOP166	Δ*tftA* (Npun_F3584)
**plasmid name**	**description**
pDDR467	Suicide vector for in-frame deletion of *tftA*
pDDR542	Shuttle vector containing *tftA* and the 301 bp 5’ to the start codon
pDDR561	pET28a containing *tftA* to express a TftA-6xHis fusion
**Primer name**	**sequence**
NpF3584-5'-F	atataggatccATGTTTTACCTTTGCTAAAAAGTTC
NpF3584-5'-R	gaagtcctgcACGTCCCATTTTCAGTATTTC
NpF3584-3'-F	gaaaatgggacgtGCAGGACTTCTTTGAGTG
NpF3584-3'-R	atatagagctcCGATCGCTTTTACATCAAGG
PNpF3584-5'-F	atataggatccTGACGGTGAAACTAGGAG
NpF3584-R	atatagagctcggTCAAAGAAGTCCTGCTGATTC
NpF3584-NcoI-F	atataccatgggcGGACGTATTTTTATATCAGCCGCTC
NpF3584-XhoI-R	atatactcgagAAGAAGTCCTGCTGATTCATATAATC
NpF3584-5'-con-F	ACCACTACTGCTTGATCTAG
NpF3584-3'-con-R	GTGTAGATGAATGGCGAATG
